# Inferior glenohumeral dislocation in an elderly

**DOI:** 10.11604/pamj.2022.41.277.29489

**Published:** 2022-04-06

**Authors:** Sakshi Pritam Arora, Waqar Mohsin Naqvi

**Affiliations:** 1 Department of Community Health Physiotherapy, Ravi Nair Physiotherapy College, Datta Meghe Institute of Medical Sciences, Wardha, Maharashtra, India

**Keywords:** Inferior dislocation, luxatio erecta, Parkinson disease, radiology, X-ray, physiotherapy

## Image in medicine

Inferior dislocation of shoulder in an elderly is the least common form of shoulder dislocation. The mechanism of injury in elderly involves forceful hyperabduction of arm over the acromion repetitively, injuring the inferior glenoid labrum and subsequently allowing the head of humerus surpass the glenoid fossa inferiorly. The diagnostic procedure involves palpation of humeral head in axilla, confirming it with radiological diagnostic imaging. The management for elderly includes immobilization of the shoulder in sling with muscle relaxants and physiotherapy. An 80-year-old female patient, a known case of stage 5-Parkinson disease (Hoehn and Yahr scale) was observed with an inevitable bulge against the ribs along the right side with ecchymosis over the right shoulder joint. In the debilitating and deteriorating state of comorbidity, she had severe stiffness which made her bed dependent and wheelchair bound. Also, she was presenting hallucination or delusions making her mobility difficult for care-taker. The luxatio erecta/inferior glenohumeral joint dislocation occurred while shifting the patient from bed to wheelchair. The patient then underwent radiological diagnosis which demonstrated luxatio erecta with neurovascular injury owing to its close proximity to axillary artery and brachial plexus. Considering the age and Parkinson disease, the dislocation was managed by closed reduction and immobilization for 6-weeks. She was then prescribed physiotherapy wherein pain was subsided with cryotherapy; stiffness and restricted range of motion were regained with passive and active mobility exercises; and strength was restored by using progressive training for 6-weeks following 40-minutes per session for 5-days a week.

**Figure 1:**
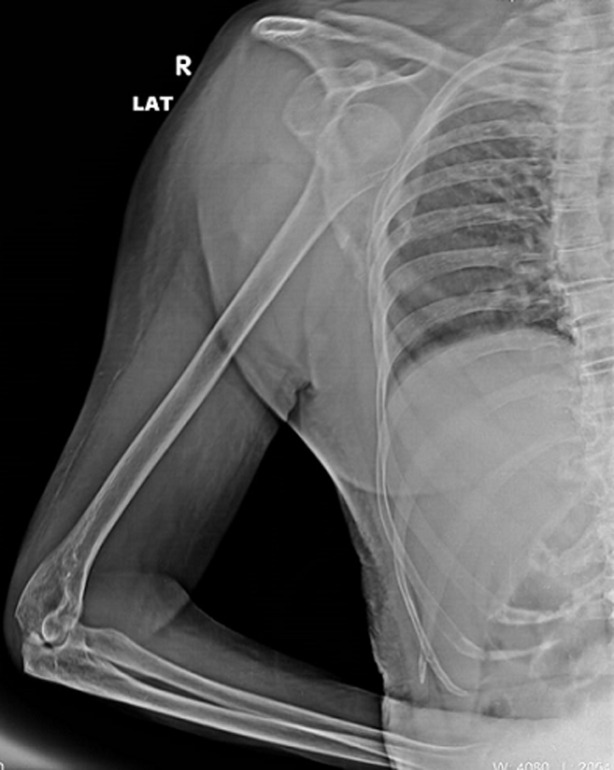
luxatio erecta or inferior glenohumeral joint dislocation

